# Array-based molecular karyotyping in fetuses with isolated brain malformations identifies disease-causing CNVs

**DOI:** 10.1186/s11689-016-9144-y

**Published:** 2016-04-15

**Authors:** Madita Schumann, Andrea Hofmann, Sophia K. Krutzke, Alina C. Hilger, Florian Marsch, Dietlinde Stienen, Ulrich Gembruch, Michael Ludwig, Waltraut M. Merz, Heiko Reutter

**Affiliations:** Institute of Human Genetics, University of Bonn, Bonn, Germany; Department of Genomics, Life and Brain Center, University of Bonn, Bonn, Germany; Department of Obstetrics and Prenatal Medicine, University of Bonn Medical School, Bonn, Germany; Department of Clinical Chemistry and Clinical Pharmacology, University of Bonn, Bonn, Germany; Department of Neonatology and Pediatric Intensive Care & Institute of Human Genetics, University of Bonn, Sigmund-Freud-Str. 25, D-53127 Bonn, Germany

**Keywords:** Array-based karyotyping, Brain malformation, Copy number variation (CNV), De novo occurrence

## Abstract

**Background:**

The overall birth prevalence for congenital malformations of the central nervous system (CNS) among Europeans may be as high as 1 in 100 live births. The etiological factors remain largely unknown. The aim of this study was to detect causative copy number variations (CNVs) in fetuses of terminated pregnancies with prenatally detected isolated brain malformations.

**Methods:**

Array-based molecular karyotyping was performed in a cohort of 35 terminated fetuses with isolated CNS malformations. Identified putative disease-causing CNVs were confirmed using quantitative polymerase chain reaction or multiplex ligation-dependent probe amplification.

**Results:**

Based on their de novo occurrence and/or their established association with congenital brain malformations, we detected five disease-causing CNVs in four fetuses involving chromosomal regions 6p25.1-6p25.3 (*FOXC1*), 6q27, 16p12.3, Xp22.2-Xp22.32 (*MID1*), and Xp22.32-Xp22.33. Furthermore, we detected a probably disease-causing CNV involving chromosomal region 3p26.3 in one fetus, and in addition, we detected 12 CNVs in nine fetuses of unknown clinical significance. All CNVs except for two were absent in 1307 healthy in-house controls (frequency <0.0008). Each of the two CNVs present in in-house controls was present only once (frequency = 0.0008). Furthermore, our data suggests the involvement of *CNTN6* and *KLHL15* in the etiology of agenesis of the corpus callosum, the involvement of *RASD1* and *PTPRD* in Dandy-Walker malformation, and the involvement of *ERMARD* in ventriculomegaly.

**Conclusions:**

Our study suggests that CNVs play an important role in the etiology of isolated brain malformations.

## Background

Congenital malformations of the brain can either be isolated or non-isolated and can affect both the neural tube and/or the brain. The overall birth prevalence among Europeans may be as high as 1 in 100 live births [1, 2]. Malformations of the brain represent a broad clinical spectrum, ranging from thinning of the corpus callosum to isolated partial or complete agenesis of corpus callosum or to alobar holoprosencephaly with complete absence of the corpus callosum [3]. These malformations may present in isolation, in association with other anomalies or as part of complex genetic syndromes. Prenatal imaging by ultrasound and magnetic resonance imaging (MRI) nowadays allows detection of most of these malformations. Based on the assumption of an impaired neurocognitive outcome, prenatal diagnosis often leads to termination of pregnancy (ToP) [4]. The underlying causes are extremely heterogeneous. Among the periconceptional or pregnancy-associated environmental risk factors are maternal intake of medications (e.g., valproate), embryonic or fetal infections (viral or parasitic, e.g., rubella, cytomegaly, or toxoplasma gondii), or maternal folic acid deficiency [5]. Among the known genetic factors are chromosomal abnormalities (e.g., trisomy 13, 18, Miller-Dieker lissencephaly syndrome [MIM #247200]) or monogenic syndromes (e.g., Joubert syndrome or holoprosencephaly type 3) [6]. However, for the majority of brain malformations, the underlying cause remains unknown.

Recent reports on the systematic investigation of underlying copy number variations (CNVs) suggest disease-causing CNVs (losses or gains of haploinsufficient or triplosensitive genomic material), to play an important role in the etiology [7–11]. In this respect, our own group recently investigated a larger cohort of fetuses from terminated pregnancies with prenatally detected syndromic brain malformations (brain malformations and additional extra-cerebral anomalies) [12]. In that cohort, we detected seven disease-causing CNVs and four probably disease-causing CNVs that were not present in our 1307 healthy in-house controls (frequency <0.0008).

Here, we report screening of 35 terminated fetuses with prenatally detected isolated brain malformations, using an array-based genome-wide approach to search for causative CNVs.

## Methods

### Subjects and DNA isolation

This study was approved by the local ethics committee, and all women provided written informed consent. The primary fetal study cohort was sampled between 2006 and 2012 through the Department of Obstetrics and Prenatal Medicine at the University of Bonn. During this time, fetal blood samples of over 400 fetuses were collected in the context of fetocide. In all cases, ToP was carried out in accordance with the German legislation. Among these fetuses, 35 presented with isolated brain malformations. Fetuses with additional extra-cerebral anomalies such as heart defects, renal anomalies, or skeletal dysplasia were excluded from the analyses. Also, fetuses with numeric chromosomal aberrations or prenatally detected monogenic syndromes were excluded.

In two of 35 fetuses, DNA quality was insufficient for array-based molecular karyotyping. Saliva samples from parents were obtained after discharge from hospital. For 10 of the 33 fetuses with sufficient DNA quality, both parents participated. Isolation of genomic DNA was carried out using either the QIAmp DNA Blood Kit (Qiagen, Hilden, Germany) or Chemagic Magnetic Separation Module I (Chemagen, Baesweiler, Germany). In the case of saliva samples, the Oragene DNA Kit (DNA Genotek Inc, Ontario, Canada) was used. For candidate gene deletion screening, we analyzed 19 additional fetuses with syndromic non-isolated Dandy-Walker malformation related brain anomalies of whom no array-based molecular karyotyping had been previously performed and which had also been sampled through the Department of Obstetrics and Prenatal Medicine at the University of Bonn.

### Prenatal conventional karyotyping

Of the 35 fetuses, 33 had sufficient DNA quality for array-based analysis. Of these 33 fetuses, only prenatal conventional karyotyping had been performed in 28 fetuses, one had FISH analysis only. In one fetus prenatal, conventional karyotyping and CGH array were performed. Here, a microscopically visible structural chromosomal aberration had been detected showing deletion of chromosomal region Xp22.32-p22.2 (fetus 14; see Table 1). Conventional prenatal karyotyping of the remaining 28 fetuses and the FISH analysis in one fetus gave normal results. The two fetuses with insufficient DNA quality for array-based molecular karyotyping had also prenatal conventional karyotyping showing normal results.

**Table 1:** Fetal phenotype and genetic findings

**Fetus genetic findings**	**Fetus 14**		**Fetus 1**	**Fetus 2**		**Fetus 3**	**Fetus 4**	**Fetus 5**	**Fetus 6**	
	Female		Male	Female		Male	Male	Male	Female	
Rearrangement (size in Mb)	delXp22.2-Xp23.2 (7.2)	dupXp22.33-Xp22.32 (1.93)	del6q27 (0.78)	dup2q32.1-2q32.2 (0.39)	del16q12.11 (0.10)	dup3q26.1 (0.51)	del3p26.3 (0.07)	del4q31.23 (0.18)	dup4q13.3 (0.25)	dup8q24.3 (0.09)
De novo	Yes	Yes	Yes	n.t.	n.t.	n.t.	n.t.	n.t.	n.t.	n.t.
1st and last Mutated SNP	rs5915786	rs5916528	rs9383520	rs7424417	rs4967746	rs4680608	rs9840732	rs17023845	rs1381015	rs7819263
	rs5978478	rs7881910	rs12530134	rs997277	rs16952589	rs2863381	rs9856251	rs13129809	rs10028486	rs10112201
Flanking SNPs	rs6530416	Telomer	rs9478086	rs7574262	rs12930613	rs1355538	rs3843386	rs6845560	rs11941162	rs3824233
	rs1526798	rs7391672	Telomer	rs1516445	rs11863453	rs10936492	rs172171	rs10025443	rs714825	rs6578185
RefSeq genes affected	23	10	13	2	1	1	1	1	2	2
**Phenotypic findings**	Callosal agenesis		Isolated symmetric internal hydrocephalus	Isolated symmetric internal hydrocephalus		ACC, colpocephaly and missing septum pellucidi	ACC, hydrocephalus, hypoplastic cerebellum, suspected lissencephaly	Macrocephaly, internal hydrocephalus	Alobar holoprosencephaly	
Prenatal karyotyping	Yes, microdeletion Xp22.2-p23.2		Yes, no results available	Yes, normal		Yes, normal	Yes, normal	Yes, normal	Yes, normal	
**Fetus genetic findings**	**Fetus 7**		**Fetus 8**	**Fetus 9**		**Fetus 10**	**Fetus 11**	**Fetus 12**	**Fetus 13**	
	Female		Male	Male		Female	Female	Male	Male	
Rearrangement (size in Mb)	dup9p23 (1.25)		del18p11.21 (0.19)	dup2q37.3 (0.10)	dup3q13.32 (0.08)	dup17p11.2-17p12 (2,43)	del16p12.2 (0.57)	del6p25.1-6p25.3 (4,65)	dupXp22.11 (0.21)	
De novo	n.t.		n.t.	n.t.	n.t.	n.t.	No paternal inheritance	Yes	Maternal	
1st and last mutated SNP	rs12552479		rs522631	rs4676385	rs4687836	rs4792576	rs670841	rs6930285	rs5925934	
	rs1335475		rs1592643	rs2288750	rs2917080	rs4646341	rs8062140	rs808601	rs10521917	
Flanking SNPs	rs1441400		rs496485	rs2975778	rs9878706	rs9907064	rs8054407	Telomer	rs7879340	
	rs7038987		rs9960249	rs4234121	rs2903301	rs12449964	rs9928431	rs3804547	rs6526366	
RefSeq genes affected	2		1	2	1	42	7	35	4	
**Phenotypic findings**	DWM, microcephaly, hydrocephalus		Occlusive hydrocephalus	Internal hydrocephalus		DWM	ACC, asymmetry of the ventricles, hypoplastic cerebellum, interhemispheric cysts, median shift	DWM	ACC	
Prenatal karyotyping	Yes, normal		No	Yes, no results available		Only FISH analysis, normal	Yes, normal	Yes, normal	Yes, normal	

*ACC* agenesis of the corpus callosum, *DWM* Dandy-Walker malformation, *FISH* fluorescence in situ hybridization, *n.t*. not tested

### Array-based molecular karyotyping

For array-based molecular karyotyping, we used the Illumina HumanOmniExpress-12 v1.0 BeadChip (marker content 730,525; median marker spacing 2.1 kb; Illumina. Inc., San Diego, CA). All analyses were performed according to the manufacturer’s protocol. A DNA sample was considered to have failed if <95 % of the SNP markers were called on the corresponding BeadChip. CNVs were predicted using the program QuantiSNP (v2.2, www.well.ox.ac.uk/QuantiSNP/) which applies an Objective-Bayes Hidden-Markov model [13].

### CNV filtering

Initial filtering of CNV data was carried out using the following seven criteria to exclude CNVs: (I) QuantiSNP quality measurement with a “log Bayes factor” <30; (II) regions with less than three aberrant markers; and (III) frequency of >1 % in our in-house control cohort (*n* = 1307 healthy controls). A series of manual filter steps was then applied: In manual step (IV), all remaining CNVs were checked visually in GenomeStudio v2011.2 (Illumina, San Diego, CA). (V) All remaining CNVs were filtered for RefSeq gene content (coding regions only, http://www.ncbi.nlm.nih.gov/refseq/rsg/) using the UCSC genome browser assembly hg19 (http://genome.ucsc.edu). CNVs that did not harbor central nervous system (CNS)-associated or CNS-expressed genes listed in Mouse Genome Informatics (MGI) or Expert Protein Analysis System (ExPASy) were excluded. In manual step (VI), CNVs were filtered according to their presence in healthy control persons in the publicly available database of Genomic Variants (DGV, http://dgvbeta.tcag.ca/dgv/app/home?ref=NCBI36/hg19). CNVs with at least five fully overlapping reports in DGV were excluded. In manual step (VII), the remaining CNVs were re-evaluated visually using the GenomeStudio genotyping module (v2011.2, http://www.illumina.com/) to check whether the breakpoints had been called correctly by QuantiSNP. If another brain-related gene may have been affected, we included the flanking region in the quantitative polymerase chain reaction (qPCR) validation. All data are designated according to hg19.

### Validation with quantitative polymerase chain reaction

The qPCR analyses were performed to confirm all putative fetal CNVs and for parental segregation analyses to detect de novo events. Reactions were performed on an ABI Prism 7900HT Fast Real-Time PCR System with SYBR Green (Applied Biosystems, Foster City, CA), using three to four primer pairs per putative CNV as described elsewhere [14]. Primer sequences are obtainable on request.

### Multiplex ligation-dependent probe amplification for CNV confirmation and cohort screening

For CNV confirmation of the *FOXC1* containing CNV, multiplex ligation-dependent probe amplification (MLPA) analysis was performed using the SALSA MLPA probemix P208-C1 Human Telomere-6 (MRC-Holland, Amsterdam, Netherlands). For cohort screening of additional fetuses, we used SALSA MLPA P054 FOXL2-TWIST1 probemix containing probes for *FOXC1* (MRC-Holland, Amsterdam, Netherlands).

## Results

For 33 fetal samples, the array quality was sufficient for analysis. In these samples, QuantiSNP called 4569 putative CNVs. After the application of filter criteria I–IV, 48 CNVs remained. The application of filter criteria V–VI decreased this number to 18, comprising ten duplications and eight deletions in a total of 14 fetuses (Table 1). Sizes ranged from 0.07 to 9.23 Mb. Ten fetuses carried a single change, and four fetuses carried two (both duplication and deletion). Seventeen of the 18 CNVs were submicroscopic, and one had already been identified with prenatal conventional karyotyping (see above; fetus 14). Sixteen of the 17 submicroscopic CNVs identified in fetuses were confirmed using quantitative PCR (qPCR) and one using multiplex ligation-dependent probe amplification. In three fetuses (fetuses 1, 12, and 14) the de novo origin of four CNVs could be confirmed. In a further fetus (fetus 11), paternal inheritance could be excluded; however, maternal DNA quality was insufficient and further sampling was declined. In another male fetus (fetus 13), maternal inheritance of a dupXp22.11 was confirmed. In the remaining 10 fetuses, CNVs could not be investigated for inheritance, as no parental DNA was available. Sixteen of the identified changes were absent in any of our 1307 healthy in-house controls (frequency <0.0008); two of the identified changes, both in fetus 9, were each present once in our 1307 healthy in-house controls (frequency = 0.0008).

### Fetuses without prenatal conventional karyotyping

*Fetus 1* (GA [gestational age, in completed weeks plus days] 25 + 2) was diagnosed by prenatal ultrasound with symmetric bilateral ventriculomegaly. The fetus carried a 0.78-Mb deletion of chromosomal region 6q27 (Chr6:170,140,184-170,919,470) comprising 13 RefSeq genes. qPCR analyses in fetus and the parents showed a de novo occurrence of the deletion in the fetus. Overlapping deletions have been previously observed in patients with brain anomalies [15].

*Fetus 8* (GA 24 + 3) was diagnosed by prenatal ultrasound with ventriculomegaly of all four ventricles and secondary macrocephaly. The fetus carried a 0.19-Mb deletion of chromosomal region 18p11.21 (chr18:12,456,559-12,643,193) comprising most of the spire-type actin nucleation factor 1 gene (*SPIRE1*, MIM 609216).

*Fetus 10* (GA 22 + 6) was diagnosed by prenatal ultrasound with Dandy-Walker variant (hypoplasia of the vermis cerebelli). The fetus carried a 2.43-Mb duplication of chromosomal region 17p11.2-17p12 (chr17:15,063,832-17,493,810) involving 42 RefSeq genes. The proximal part of this duplication has been associated with Potocki-Lupski syndrome [16], resulting from variable duplications at 17p11.2.

### Fetuses with prenatal conventional karyotyping

*Fetus 2* (GA 25 + 6) was diagnosed by prenatal ultrasound with isolated, symmetric bilateral ventriculomegaly. The fetus carried a 0.39-Mb duplication of chromosomal region 2q32.1-2q32.2 (chr2:189,363,100-189,750,863) comprising the complete coding regions of the two RefSeq genes *GULP1* (PTB domain containing engulfment adaptor protein 1, MIM 608165) and *DIRC1* (mRNA disrupted in renal carcinoma 1, MIM 606423). This fetus carried an additional 0.1-Mb deletion of chromosomal region 16q12.11 (chr16:47,074,235-47,174,275) comprising exons 2–9 of the *NETO2* gene (neuropilin and tolloid (TLL)-like 2, MIM 607974).

*Fetus 3* (GA 12 + 6) was diagnosed by prenatal ultrasound with agenesis of the corpus callosum (ACC) (colpocephaly and absent septum pellucidum). The fetus carried a 0.51-Mb duplication of chromosomal region 3q26.1 (chr3:165,553,646-166,065,099) affecting *BCHE* gene exon 1 (butyrylcholinesterase, MIM 177400).

*Fetus 4* (GA 24 + 0) was diagnosed by prenatal ultrasound with symmetric bilateral ventriculomegaly, cerebellar hypoplasia, ACC, and signs of lissencephaly. The fetus carried a 0.07-Mb deletion of chromosomal region 3p26.3 (chr3:1,436,664-1,511,108), comprising the last three exons (21–23) of the *CNTN6* gene (contactin 6, MIM 607220). CNVs comprising *CNTN6* have recently been associated with neurodevelopmental discorders and brain anomalies [17, 18].

*Fetus 5* (GA 21 + 3) was diagnosed by prenatal ultrasound with occlusive hydrocephalus (with dilatation of the lateral and third ventricles, suggesting aquaeductal stenosis) and secondary macrocephaly. The fetus carried a 0.18-Mb deletion of chromosomal region 4q31.23 (chr4:148,727,355-148,903,388), comprising the single RefSeq gene *ARHGAP10* (Rho GTPase activating protein 10, MIM 609746).

*Fetus 6* (GA 23 + 4) was diagnosed by prenatal ultrasound with alobar holoprosencephaly. The fetus carried two duplications. The first duplication was 0.25 Mb in size affecting chromosomal region 4q13.3 (chr4:73,812,806-74,058,406) comprising the complete coding region of the *COX18* gene (cytochrome c oxidase assembly factor, MIM 610428) and part of the *ANKRD17* gene (ankyrin repeat domain 17, MIM 615929). The second duplication was 0.09 Mb in size affecting chromosomal region 8q24.3 (chr8:142,295,965-142,382,757). The region comprises the complete *GPR20* gene (G protein-coupled receptor 20, MIM 601908) and the long non-coding RNA *LOC7311779*.

*Fetus 7* (GA 27 + 5) was diagnosed by prenatal ultrasound with Dandy-Walker malformation (agenesis of the vermis cerebelli) with bilateral symmetric ventriculomegaly and microcephaly. The fetus carried a 1.25-Mb duplication of chromosomal region 9p23 (chr9:10,144,584-11,398,305) affecting the *PTPRD* gene (protein-tyrosine phosphatase, receptor-type, delta, MIM 601598) and its complete long non-coding RNA, antisense RNA 2 (head to head) (*PTPRD-AS2*).

*Fetus 9* (GA 21 + 6) was diagnosed by prenatal ultrasound with symmetric bilateral ventriculomegaly and secondary macrocephaly. This fetus carried two duplications. The first duplication was 0.1 Mb in size affecting chromosomal region 2q37.3 (chr2:241,623,894-241,722,445) comprising the two RefSeq genes *KIF1A* (kinesin family member 1A, MIM 601255) and *AQP12A* (aquaporin 12A, MIM 609789). Here, the *KIF1A* gene has been associated with hereditary sensory and autonomic neuropathy type II and in autosomal recessive spastic paraparesis as well as spastic paraplegia [19–21]. The second duplication was 0.08 Mb in size affecting chromosomal region 3q13.32 (chr3:118,730,933-118,812,027) comprising intron 3 of the *IGSF11* gene (immunoglobulin superfamily, member 11, MIM 608351).

*Fetus 11* (GA 28 + 3) was diagnosed by prenatal ultrasound with ACC (colpocephaly and absent septum pellucidum) and interhemispheric arachnoidal cysts. This fetus carried a 0.57-Mb deletion of chromosomal region 16p12.2 (chr16:21,839,340-22,409,463). This region comprises seven RefSeq genes namely *UQCRC2* (ubiquinol-cytochrome c reductase core protein II, MIM *191329*), *PDZD9* (PDZ domain containing 9), *C16orf52*, *VWA3A* (von Willebrand factor A domain containing 3A), *EEF2K* (eukaryotic elongation factor 2 kinase, MIM 606968), *POLR3E* (polymerase (RNA) III (DNA directed) polypeptide E (80kD)), and *CDR2* (cerebellar degeneration-related protein 2, MIM 117340).

*Fetus 12* (GA 22 + 3) was diagnosed by prenatal ultrasound with Dandy-Walker malformation (agenesis of the vermis cerebelli with bilateral, symmetric ventriculomegaly and secondary macrocephaly). The fetus carried a 4.65-Mb deletion of chromosomal region 6p25.1-6p25.3 (chr6:212,548-4,864,581) comprising 35 RefSeq genes. CNV validation via MLPA confirmed the deletion and analysis of parental DNA established its de novo occurrence. Among the deleted genes resides *FOXC1* (forkhead box C1, MIM 601090). Point mutations and deletions of *FOXC1* have been shown to play a major role in the formation of Dandy-Walker malformations [22, 23].

Prompted by our finding of a deletion in fetus 12 comprising *FOXC1*, we used a region-specific high-density MLPA probe (see above) to screen 19 additional fetuses with syndromic non-isolated Dandy-Walker malformation-related brain anomalies of whom no array-based molecular karyotyping had been previously performed. However, this additional analysis failed to detect any further deletions (or duplications) of the *FOXC1* region.

*Fetus 13* (GA 23 + 4) was diagnosed by prenatal ultrasound with ACC. The fetus carried a 0.21-Mb duplication of chromosomal region Xp22.11 (chrX:23,799,472-24,012,381). The region comprises four RefSeq genes encoding *SAT1* (spermidine/spermine N1-acetyltransferase, MIM 313020), *APOO* (apolipoprotein O, MIM 300753), *CX0rf58*, and *KLHL15* (kelch-like family member 15). Interestingly, *KLHL15* was most recently identified as a potential X-linked intellectual disability (ID) gene [24]. Investigation of parental DNA showed transmittance of this CNV from the healthy mother.

*Fetus 14* (GA 28 + 5) was diagnosed by prenatal ultrasound with ACC. The female fetus carried a 7.2-Mb deletion of chromosomal region Xp22.2-Xp22.32 (chrX:4,642,016-11,935,042) comprising 23 RefSeq genes. Among these genes is the midline 1 (*MID1*) gene. Mutations in *MID1* have been associated with the X-linked form of Opitz syndrome [25], which is characterized by midline abnormalities such as cleft lip, laryngeal cleft, heart defects, hypospadias, and ACC [26]. Furthermore, and on the same X-chromosome, the fetus carried a 1.93-Mb duplication, involving chromosomal region Xp22.32-Xp22.33 (chrX:2,700,157-4,628,697), comprising the following 12 RefSeq genes: *GYG2* (glycogenin 2, MIM 300198), *XG* (Xg blood group, MIM 300879), *ARSD* (arylsulfatase D, MIM 300002), *ARSE*, *ARSH*, *ARSF* (arylsulfatases E, H, and F, MIM 300180, 300586, 300003), *MXRA5* (matrix-remodeling associated 5, MIM 300938), *PRKX* (protein kinase, X-linked, MIM 300083) and its long non-coding RNA (*PRKX-AS1*), long intergenic non-protein coding RNA 1546 (*LINC01546*), and the uncharacterized long non-coding RNAs *LOC101928201* and *LOC389906*. qPCR CNV validation and segregation analysis of the fetus and the parents confirmed both the deletion and the duplication and revealed their de novo occurrence in the fetus.

## Discussion

During embryonic and fetal life, the CNS forms and later matures in a complex developmental process. Milestones in early gestation are pattern formation (induction of the neurectoderm and segmentation of the neural tube) and morphogenetic events like neurulation, resulting in the folding of the neural tube and prosencephalon. Starting in mid-gestation, neuronal proliferation, migration, organization, and myelination are subsequent important steps. These events and their respective timing are governed by a complex interaction of a multitude of genes. The present study applied array-based molecular karyotyping in fetuses with isolated brain malformations to search for novel causative CNVs. Based on their de novo occurrence and/or their established association with congenital brain malformations, we detected five disease-causing CNVs in four fetuses (fetus 1, fetus 10, fetus 12, and twice in Fetus 14). Furthermore, we detected a probably disease-causing CNV involving chromosomal region 3p26.3 in one fetus (fetus 4) and, in addition, we detected 12 CNVs in nine fetuses (fetus 2, fetus 3, fetus 5, fetus 6, fetus 7, fetus 8, fetus 9, fetus 11, and fetus 13) of unknown clinical significance. All CNVs, except for two, were absent in 1307 healthy in-house controls (frequency <0.0008). Each of the two CNVs present in in-house controls was present only once (frequency = 0.0008). In the following, we will discuss the detected CNVs in the context of their associated phenotype.

### Dandy-Walker malformation

Dandy-Walker malformation (DWM; OMIM %220200) is a heterogeneous disorder, defined by elevated tentorium and torcular, upward rotation of the vermian axis, and agenesis or partial agenesis of the cerebellar vermis with cystic dilation of the fourth ventricle, sometimes associated with secondary macrocephaly. Variable hypoplasia of cerebellar vermis with and without enlargement of the posterior fossa is named DWM variant [27, 28]. Previously, maternally transmitted CNVs (dup9p21.2, dup10p15.3) were described in two DWM patients [7], whereas *FOXC1* deletions [23] and single gene mutations for *FOXC1* (MIM 601090) [22], *ZIC1* (MIM 600470), and *ZIC4* (MIM 608948) [29] were identified in several DWM patients. Here, isolated DWM was observed in two of our fetuses (fetuses 10 and 12) and in fetus 7 with additional microcephaly and bilateral ventriculomegaly.

Fetus 7, diagnosed with DWM, carried a 1.25-Mb duplication of chromosomal region 9p23, affecting parts of the *PTPRD* gene (MIM 601598) and its complete long non-coding RNA (*PTPRD-AS2*). *PTPRD* is expressed in different brain regions, acting as an important regulator of synaptic plasticity and synapse organization [30, 31]. Although duplication in 9p23 has not been reported in association with congenital brain malformation, a smaller dup9p23, affecting solely *PTPRD* has been detected in a patient with bipolar disorder [32]. In our fetus, the duplication of *PTPRD-AS2*, which may be involved in *PTPRD* mRNA regulation, may contribute to an unbalanced rate of protein production, thereby leading to the non-isolated DMW observed.

Fetus 10 carried a 2.43-Mb duplication of chromosomal region 17p11.2-17p12. The proximal part of this duplication has been associated with a neurobehavioral phenotype in the Potocki-Lupski syndrome [16], resulting from variable duplications at 17p11.2. Zhang et al. [33] reviewed 74 duplications involving 17p11.2 and identified a smallest region of overlap, solely including the *RAI1* (retinoic acid inducible 1) gene. The duplication detected in fetus 10 and at least in one DECIPHER patient (accession number #274909) with microcephaly did not affect *RAI1*. Hence, another gene(s) might be responsible for DWM in our case. Among the 42 affected genes in our fetus is *RASD1*, encoding the small GTPase dexamethasone-induced Ras-related protein 1. RASD1 was shown to be a protein with enriched expression in brain, specifically coupled to neuronal nitric oxide synthase [34]. No embryonic expression data have been published; however, RASD1 has yet been localized to the suprachiasmatic nucleus, thalamus, piriform cortex, and the hippocampus in adult mice and in rat vasopressin neurons of the supraoptic nucleus and paraventricular nucleus [35, 36]. As most recently outlined by Chen and colleagues [37], RASD1 plays a central role in neuronal iron trafficking and the regulation of adenylyl cyclase and G-protein-linked neurotransmitter, making it an attractive candidate for DVM. However, it cannot be excluded that even genes outside this CNV may be affected by the duplication event.

Fetus 12 carried a 4.65-Mb deletion involving chromosomal region 6p25.1-6p25.3 comprising *FOXC1*. Hence, the deletion in our fetus 12 supports the findings of Aldinger et al. [22] and Delahaye et al. [23] that *FOXC1* is a major contributor to DWM and should explain the observed phenotype. In summary, *RASD1* and *PTPRD* might be further contributors to the etiology of DWM.

### Agenesis of the corpus callosum

Agenesis of the corpus callosum (ACC; MIM %217990) is a clinically and genetically heterogeneous disorder [38]. Various CNVs have been observed in association with ACC [7, 10, 38] and also mutations in several genes [7, 38]. Here, we detected CNVs in five fetuses (fetuses 3, 4, 11, 13, and 14).

Fetus 3, diagnosed with ACC, carried a novel 0.51-Mb duplication affecting exon 1 of the *BCHE* gene on 3q26.1. Soreq et al. [39] implicated this gene in cholinergic influence on cell growth and proliferation. The Mouse Gene Expression Database (http://www.informatics.jax.org/expression.shtml) reports *Bche* expression in the brain. Whether *BCHE* is a candidate gene for brain malformations remains to be elucidated.

Fetus 4 carried a 0.07-Mb deletion of chromosomal region 3p26.3, solely involving the last three exons of the *CNTN6* gene. Kashevarova et al. [18] recently reported three 3p26.3 microdeletions/duplications encompassing only *CNTN6* and reviewed a total of nine further cases, all presenting with ID. Also, Te Weehi et al. [40] implicated that a duplication of 3p26.3 in cognitive development and brain anomalies were always observed in the larger deletion syndrome, affecting 3p25-pter [38, 41]. Contactins are neuronal membrane proteins that mediate cell surface interactions during nervous system development. As with other family members, *CNTN6* is suggested to be involved in the modulation of neurite outgrowth, synaptogenesis, survival guidance of projections, and terminal branching of axons [42]. Data from knockout mice showed *Cntn6* to participate in embryonic brain development and synapse formation in postnatal cerebellar development [43]. Hence, alterations in *CNTN6* dosage, caused by either deletion or duplication, may interfere with correct brain development.

Fetus 11, diagnosed with ACC, carried a 0.57-Mb deletion of chromosomal region 16p12.2. This deletion has been extensively described by Girirajan et al. [44] and is characterized by variable clinical findings with various forms of brain anomalies, ID, or developmental delay (DD) but does not constitute a recognizable syndrome. Hence, it should explain the phenotype observed in our fetus. The region harbors seven RefSeq genes. Among the deleted genes is *CDR2*. Cdr2 protein has been shown in human to interact with c-myc to synergistically regulate c-myc-dependent transcription during mitosis, and loss of Cdr2 leads to aberrant spindle formation and impaired proliferation [45]. Disturbance of this role for Cdr2 in dividing cells during mitosis may hence interfere with normal brain development.

Fetus 13, diagnosed with ACC, carried a maternally inherited 0.21-Mb duplication of chromosomal region at Xp22.1 comprising *APOO*, *SAT1*, and *KLHL15*. Several duplications of this region have been described in DECIPHER in patients with brain anomalies. Mignon-Ravix et al. [24] and most recently Hu et al. [46] suggested *KLHL15* is a novel gene involved in X-linked ID. KLHL15 protein has been shown a substrate-specific adapter of an E3 ubiquitin-protein ligase complex, which mediates ubiquitination and subsequent proteasomal degradation of target proteins, specifically targeting protein phosphatase 2A, enriched in the nervous system [47]. Duplication of *KLHL15* might hence release a dose-effect in this degradation process, leading to ACC.

Fetus 14, diagnosed with ACC, carried a de novo 7.2-Mb deletion of chromosomal region Xp22.2-Xp22.32. Among the deleted genes is *MID1*. Mutations in *MID1* have been associated with the X-linked form of Opitz syndrome [25], characterized by midline abnormalities such as cleft lip, laryngeal cleft, heart defects, hypospadias, and ACC [26]. Hence, haploinsufficiency of *MID1* may explain ACC in the fetus. Besides this deletion, fetus 14 carried also a de novo 1.93-Mb duplication of chromosomal region Xp22.32-Xp22.33. Although pure duplications of this region have rarely been reported [24], several patients listed in DECIPHER showed ID, global mental delay, and even hypoplasia of the corpus callosum (patient 278363) suggesting this duplication may also contribute to the phenotype of fetus 14.

The ACC phenotype in two of the described fetuses is most likely attributable to the deletion of *MID1* and 16p12.2. Furthermore, we suggest the involvement of *CNTN6* in ACC and propose *BCHE* as a possible candidate gene for ACC.

### Alobar holoprosencephaly

Holoprosencephaly (HPE) is the most common structural malformation of the human forebrain (prosencephalon), its most severe form being alobar HPE (aHPE). Here, there is only a single ventricle, no interhemispheric fissure, and the olfactory bulbs and tracts and the corpus callosum are typically absent. The etiology of HPE is extremely heterogeneous, and affected cases may have a recognizable monogenic syndrome, chromosomal anomalies, or mutations in one of the genes encoding *SHH*, *SIX3*, *ZIC2*, or *TGIF* [48].

Our analysis identified two duplications in fetus 6, diagnosed with HPE. The first 0.25-Mb duplication affects chromosomal region 4q13.3 and comprises the complete *COX18* gene and part of the *ANKRD*17 gene. A similar duplication has been observed in three DECIPHER patients, all showing DD or ID. *COX18* is required for the insertion of integral membrane proteins into the mitochondrial inner membrane and essential for the activity and assembly of cytochrome c oxidase. Data on ANKRD17 protein suggest that it interacts with proteins involved in DNA replication, as loss of *ANKRD17* affects Cdc6 and *PCNA* (proliferating cell nuclear antigen) loading onto DNA [49]. Interestingly, in a most recent report, Baple et al. [50] identified a homozygous missense mutation in the *PCNA* gene in four individuals from an Amish pedigree with ataxia-telangiectasia-like disorder-2 (MIM #615919), presenting with a neurodegenerative phenotype characterized by DD, ataxia, and sensorineural hearing loss. In vitro studies, as well as studies of patient cells, showed a negative effect of the mutation on nucleotide excision repair. These findings imply that a disturbed ANKRD17-PCNA interaction may also be involved in aHPE.

Fetus 6 also carried a 0.09-Mb duplication, including the complete G protein-coupled receptor 20 (*GPR20*) gene at 8q24.3. *GPR20* has been shown to be expressed in different brain regions [51] and seems to be involved in cellular processes like mitogenic signaling and the control of intracellular cAMP levels [52]. A role for *GPR20* in aHPE is supported by several DECIPHER patients with an overlapping duplication and ACC, ID, or DD phenotypes.

### Isolated or non-isolated ventriculomegaly

Ventriculomegaly is very heterogenous in nature and complex in its etiology. As outlined by Tully and Dobyns [53], it is defined as any increase in cerebrospinal fluid within the skull and more narrowly as ventricular enlargement causing accelerated head growth or requiring surgical intervention. Today, more than 10 genes have been implicated in hydrocephalus without major additional phenotypic findings, and more than 25 genes are associated with hydrocephalus and major additional physical features [53]. However, many more genes may contribute to this brain anomaly.

Here, fetus 1, diagnosed with ventriculomegaly, carried a 0.78-Mb de novo deletion of chromosomal region 6q27. Similar deletions have frequently been observed in patients with brain anomalies including ACC, hydrocephalus, periventricular nodular heterotopia, and cerebellar malformations [15]. The smallest deleted region of overlap in these previously described patients spans 1.7 Mb, containing *DLL1*, *C6orf70* (*ERMARD*), *PHF10*, and *THBS2*. Based on our findings, the newly defined smallest region of overlap excludes *PHF10* and *THBS2*. Conti et al. [54] showed data suggesting that *C6orf70*, also termed *ERMARD* (ER membrane-associated RNA degradation), plays a major role in the control of neuronal migration, and its haploinsufficiency or mutation causes periventricular nodular heterotopia. Moreover, heterozygous *ERMARD* mutations have been associated with brain anomalies and periventricular nodular heterotopia 6 (OMIM #615544) [54]. These data suggest that haploinsufficiency of *ERMARD* is a contributor to brain anomalies and should explain the symmetric internal hydrocephalus observed in our fetus.

Fetus 2, diagnosed with ventriculomegaly, carried a 0.10-Mb deletion of chromosomal region 16q12.11 and a 0.39-Mb duplication of chromosomal region 2q32.1-2q32.2. The deletion affects exons 2–9 of the *neuropilin and tolloid* (*TLL*)-*like 2* (*NETO2*) gene, and overlapping deletions were reported in two DECIPHER patients (2856, 289229) who showed, among other features, ID and global DD. In rat, Neto2 was shown to be a brain-specific protein, modulating glutamate signaling in the brain by regulation of the kainate receptor function [55, 56]. Ivakine et al. [57] revealed murine Neto2 as a neuron-specific K^+^/Cl^−^ cotransporter (Kcc2)-interacting protein required for neuronal Cl^−^ regulation in hippocampal neurons. These findings make human *NETO2* an attractive candidate gene for involvement in the etiology of hydrocephalus. The additional 0.39-Mb duplication seen in fetus 2 involves chromosomal region 2q32.1-2q32.2, comprising the complete genes *GULP1* and *DIRC1*. GULP1 is a highly evolutionarily conserved adapter protein, involved in the prompt clearance of cells undergoing apoptosis, a function critical during embryonic development, normal tissue turnover, inflammation, and autoimmunity [58]. The function of *DIRC1* is yet unclear, but there is no evidence for its expression in fetal brain. As a similar duplication has not been reported thus far, it remains to be elucidated, if at least *GULP1* may contribute to the phenotype observed.

Fetus 5, diagnosed with ventriculomegaly, carried a 0.18-Mb deletion involving most of the *ARHGAP10* gene on chromosome 4q31.23. A similar deletion, affecting only part of *ARHGAP10* has been observed in a single DECIPHER patient (280885), who showed generalized seizures. This gene encodes a cytoskeletal Rho-GTPase-activating protein with high levels of expression in the brain and muscle, and it is suggested to play an important role in cell differentiation [59].

Fetus 8, diagnosed with ventriculomegaly, carried a 0.19-Mb deletion of chromosomal region 18p11.21 comprising most of the *SPIRE1 g*ene. This region is involved in the larger 18p deletion syndrome, and most patients have either loss of large parts of 18p, up to the entire p-arm, or show a chromosomal rearrangement involving other chromosomes [60]. Monosomy 18p is often associated with brain anomalies. A correlation between the breakpoints and the mental development of seven patients led to the suggestion of a critical region between p11.1 and p11.21, since three patients with a deletion distal to this point showed normal or borderline mental development [61]. In our fetus, the deletion affected solely the *SPIRE1* gene, encoding a protein involved in actin organization. In mice, *Spire1* expression has been observed during embryogenesis in the developing nervous system and in the adult brain [62], making haploinsufficiency for *SPIRE1* an attractive contributor for brain anomalies, as well as in the context of larger 18p deletions.

Fetus 9, diagnosed with ventriculomegaly, carried two duplications. The first 0.1-Mb duplication involves chromosomal region 2q37.3, comprising *KIF1A* and *AQP12A*. The *KIF1A* gene, encoding an axonal transporter of synaptic vesicles involved in nuclear migration and neurogenesis [63], has been associated with hereditary sensory and autonomic neuropathy type II and in autosomal recessive spastic paraparesis as well as spastic paraplegia [19–21]. Most recently, Okamoto et al. [64] identified a de novo heterozygous *KIF1A* missense mutation in a patient with progressive neurodegeneration. A similar CNV as that observed in our fetus has also been deposited in DECIPHER (patient 2692) affected with ID and small stature.

The second 0.08-Mb duplication involved chromosomal region 3q13.32 and comprised the *IGSF11* gene. IGSF11 is an immunoglobulin (Ig) superfamily member, preferentially expressed in the brain and testis [65]; however, an involvement in the etiology of brain malformations has not been reported and a similar duplication has not been observed yet. According to our observation and previous reports, we suggest, at minimum, *KIF1A* as a possible disease-causing gene in brain plasticity and suggest a causal relationship with the fetal phenotype.

While we were able to identify disease-causing and probably disease-causing CNVs, our study has several limitations, inherent to its design: The fetal brain develops dynamically and depending on the gestational age, a given developmental stage may not yet have taken place. Accordingly, certain malformations manifest themselves late in gestation or postnatally, e.g., some neuronal migration disorders and septo-optic dysplasia, which are often combined with agenesis of the corpus callosum, will only be detectable by imaging techniques late in pregnancy or even postnatally. Additionally, at the time of recruitment, cerebral magnetic resonance tomography was not yet established as an adjunct for the diagnosis of fetal brain malformations. Finally, the results of neurohistologic examinations are limited due to the autolytic process occurring between fetocide and delivery.

## Conclusions

Our CNV analysis revealed disease-causing CNVs in 12 % (four out of 33 fetuses) involving chromosomal regions 6p25.1-6p25.3, 6q27, 16p12.3, Xp22.2-Xp22.32, and Xp22.32-Xp22.33. Furthermore, we detected a probably disease-causing CNV involving chromosomal region 3p26.3 and additional 12 rare CNVs (frequency ≤0.0008) in nine fetuses of unknown clinical significance. Our study suggests that CNVs play an important role in the etiology of isolated brain malformations.
